# A Case of Embolization of a Lymphatic Malformation Complicated by Chylothorax, Chylopericardium, and Interstitial Lung Disease in a 6‐Year‐Old Pediatric Patient

**DOI:** 10.1155/crra/1095827

**Published:** 2026-08-19

**Authors:** Eleonora Rossi, Duccio Rossi, Mario Petrillo, Gianpaolo Carrafiello, Andrea Antonio Ianniello

**Affiliations:** ^1^ Università degli Studi di Milano, Milan, Italy, unimi.it; ^2^ Department of Radiology, ASST Fatebenefratelli Sacco, Luigi Sacco Hospital, Milan, Italy, asst-fbf-sacco.it; ^3^ Diagnostic and Interventional Radiology, Università degli Studi di Milano, Milan, Italy, unimi.it; ^4^ IRCCS Ca′Granda Fondazione Ospedale Maggiore Policlinico, Milan, Italy, policlinico.mi.it

## Abstract

**Background:**

A 6‐year‐old child with a clinical history of chylopericardium treated with pericardiocentesis presented with retrosternal chest pain and asthenia in the past 2–3 days. The patient underwent a chest‐abdominal MRI revealing pericardial and pleural effusion with lymphatic malformation of the thoracic duct and lymphatic abdominal vessels.

**Materials and Methods:**

The patient underwent a thoracic duct embolization, consisting of two stages. At the first stage, a US‐guided catheterization of a hepatic lymphatic duct and of two inguinal lymph nodes bilaterally was carried out to perform a dynamic contrast‐enhanced MR intranodal lymphangiography (DCMRL). DCMRL showed lymphangiectasia of retroperitoneal lymphatics and of the thoracic duct with double drainage into the left subclavian vein and a possible lymphatic leak near the pulmonary veins. At a second stage, a thoracic duct catheterization and lymphography were performed, confirming the lymphatic leakage into the pericardial cavity and allowing the embolization of the thoracic duct extending to L1 vertebral level through pushable and detachable coils followed by injection of cyanoacrylate diluted 1:1 with lipiodol.

**Conclusion:**

This case was emblematic as it underlined the importance of DCMRL as a preprocedural diagnostic tool for studying lymphatic variants and malformations of patients needing any lymphatic treatment. It also added evidence on the effectiveness of this interventional approach for treating chylous effusions and interstitial lung disease.

## 1. Introduction

Lymphatic malformations (LM) include a variety of developmental abnormalities characterized by the presence of anomalous lymphatic tissue in multiple districts, including lungs, soft tissue, and bones [1, 2]. Patients with thoracic LM typically present with chylothorax and interstitial lung disease, often leading to impaired pulmonary function [1, 2].

Imaging of the lymphatic system plays a key role in assessing the anatomy of the malformation and in identifying the cause of its complications [1, 3–5]. These steps are fundamental for planning a potential interventional therapeutic approach, such as lymphatic embolization [1, 3–6].

The recent introduction of dynamic contrast‐enhanced MR intranodal lymphangiography (DCMRL) has revolutionized lymphatic imaging, which previously relied on the more complex and less efficient pedal lymphangiography and lymphoscintigraphy [1, 3]. DCMRL involves ultrasound (US) and fluoroscopy‐guided access of inguinal lymph nodes using a 25G needle, followed by the injection of gadolinium‐based contrast at a rate of 0.5–1 mL/min [1, 3].

This case‐report describes the use of DCMRL and thoracic duct (TD) embolization for treating a 6‐year‐old patient with chylothorax and chylopericardium due to lymphangiectasia of the lumbar lymphatics.

## 2. Case Presentation

We present the case of a 6‐year‐old male child who was referred to us in March 2025 for a brief episode of sleep apnea, reduced appetite, and retrosternal chest discomfort lasting 2–3 days. Vital signs were within normal limits. Physical examination revealed muffled heart sounds. Blood tests and electrocardiogram were normal. Echocardiography and cardiological consultation revealed chronic pericardial effusion (17 mm depth) without signs of cardiac tamponade. Hospital admission followed, and treatment with ibuprofen and colchicine was initiated but soon interrupted due to suspicion of a chylous pericardial effusion.

**Figure 1:**
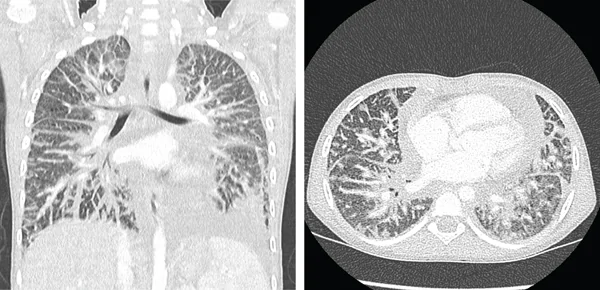
CT scan axial and coronal images showing the main pattern of ILD presented by the patient.

Chest CT scan and chest‐abdominal MRI revealed pericardial and pleural effusion, along with interstitial lung disease characterized by interstitial edema and thickening of the pulmonary fissures and interlobular septa, particularly in the middle‐inferior pulmonary lobes (Figure 1). The findings were inconclusive, as lymphangiectasia, lymphangiomatosis, or other pathological conditions could not be excluded (e.g., plastic bronchitis or Waldmann disease) [2, 3].

The patient had previously been hospitalized in Romania between February and March 2024 for cardiac tamponade secondary to chylopericardium. Presentation included dyspnea and fatigue. Echocardiography showed a pericardial effusion (depth of about 25 mm) with partial diastolic collapse of the right atrium. Physical examination was mostly unremarkable, with mild jugular venous distension. Blood tests were negative.

The patient underwent pericardiocentesis and was diagnosed with chylopericardium. Octreotide therapy was initiated. Imaging (angio‐CT scan, abdominal CT scan with contrast, and echocardiography) showed no further abnormalities.

A TD embolization was performed in two stages under general anesthesia. Institutional review board approval was obtained and patients′ informed consent was waived.

**Figure 2:**
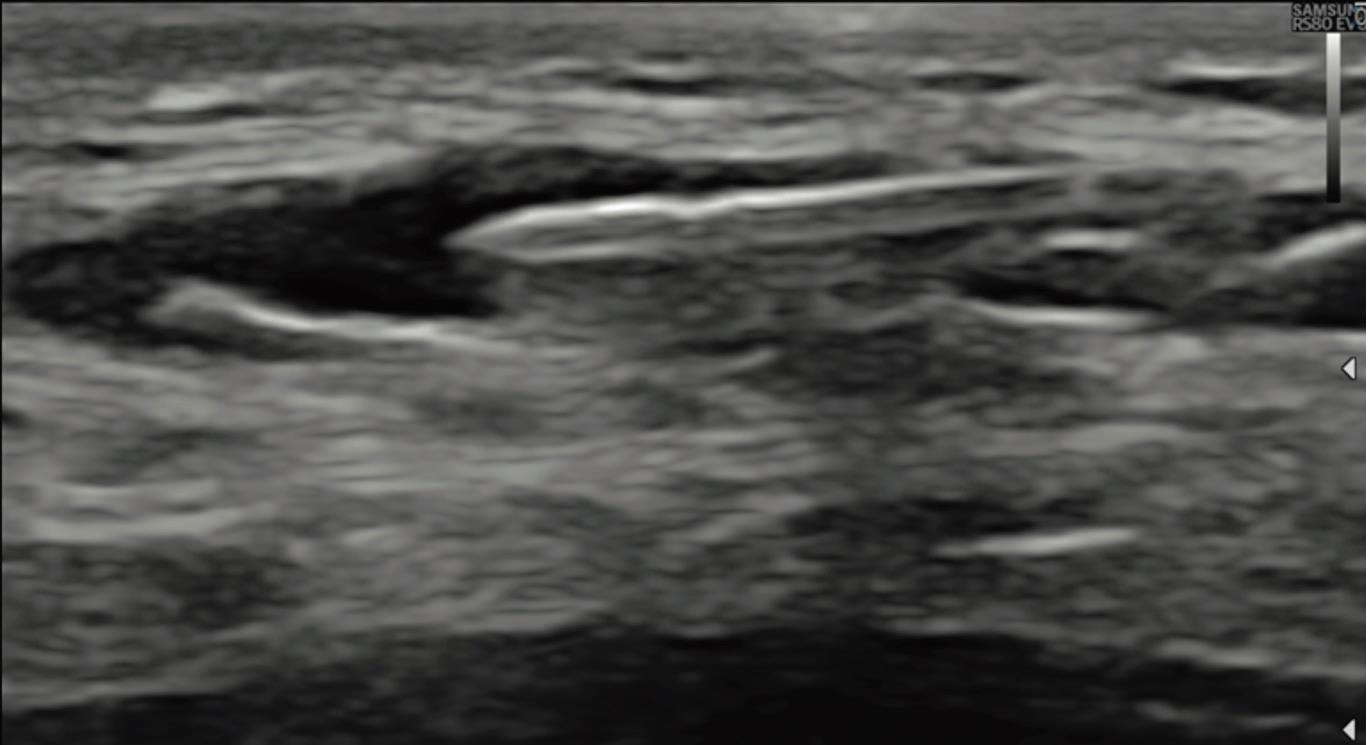
Longitudinal ultrasound image showing access of the inguinal lymph node with a 23G needle.

**Figure 3:**
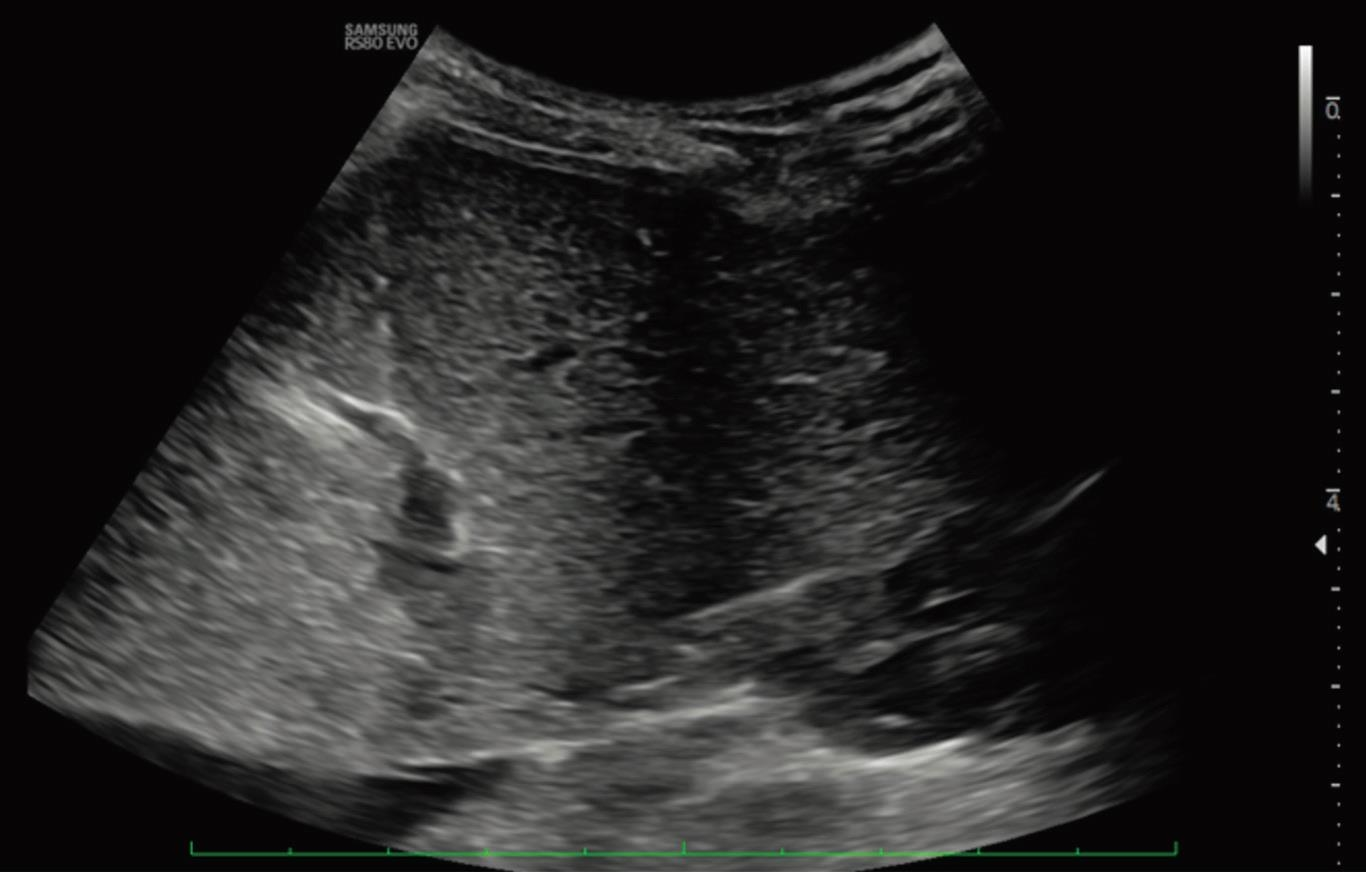
Transverse ultrasound image showing hepatic lymphatic access with a 23G needle anterior to the portal vein.

At the first stage, US‐guided catheterization of bilateral inguinal lymph nodes was performed using a 25G needle (Figure 2). Additionally, a US‐guided catheterization of a hepatic lymphatic duct near the portal vein was performed (Figure 3).

**Figure 4:**
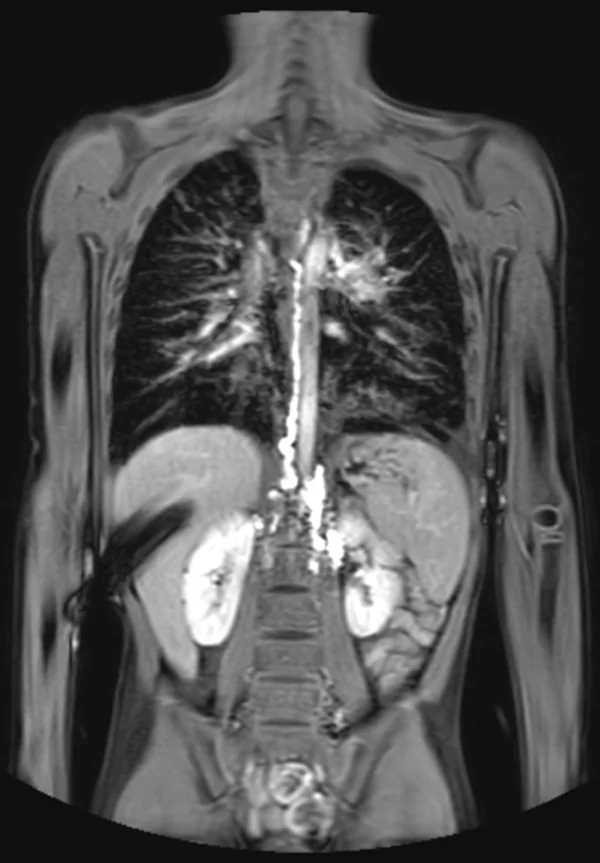
Coronal T1‐weighted postcontrast image showing a markedly tortuous thoracic duct anatomy, a crucial information to guide further therapeutic planning.

**Figure 5:**
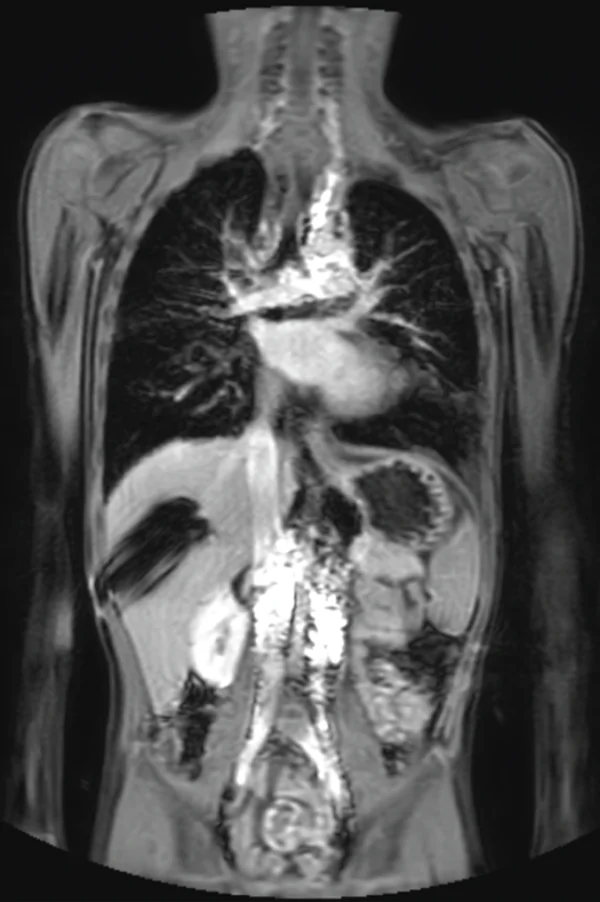
Coronal T1‐weighted postcontrast image showing an extensive lymphatic malformation extended to both the subdiaphragmatic lymphatics and the mediastinum, with a possible lymphatic leak near the pulmonary veins.

A DCMRL was then conducted after intralymphatic injection by hand of a solution of 13 mL Gadolinium diluted 1:2.5 with saline, according to guidelines [4]. The exam confirmed LM with lymphangiectasia of retroperitoneal lymphatics and TD (Figures 4 and 5), which exhibited double drainage into the left subclavian vein. A possible lymphatic leak near the pulmonary veins, likely the source of the pericardial effusion [5, 7], was also identified (Figure 5).

Compared with the previous imaging, left pleural effusion was reduced, though severe bilateral thoracic lymphangitis persisted.

At the second stage, TD catheterization was achieved with a 23G needle, after prior hepatic lymphatic duct opacification [4]. A Transend guidewire was advanced into the distal third of the TD. The 23G needle was then removed to allow the insertion of a Marathon microcatheter. The guidewire was then exchanged for a Terumo Advantage guidewire, allowing for microcatheter exchange for a Mamba Flex microcatheter to reach the terminal segment of the TD and its junction with the subclavian vein.

**Figure 6:**
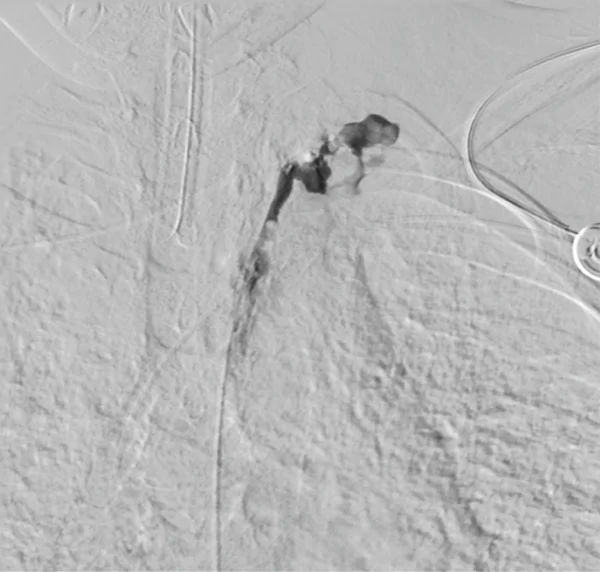
Frontal fluoroscopic image showing an antegrade access of the thoracic duct and its opacification via water‐soluble contrast, confirming the intramediastinal leakage.

**Figure 7:**
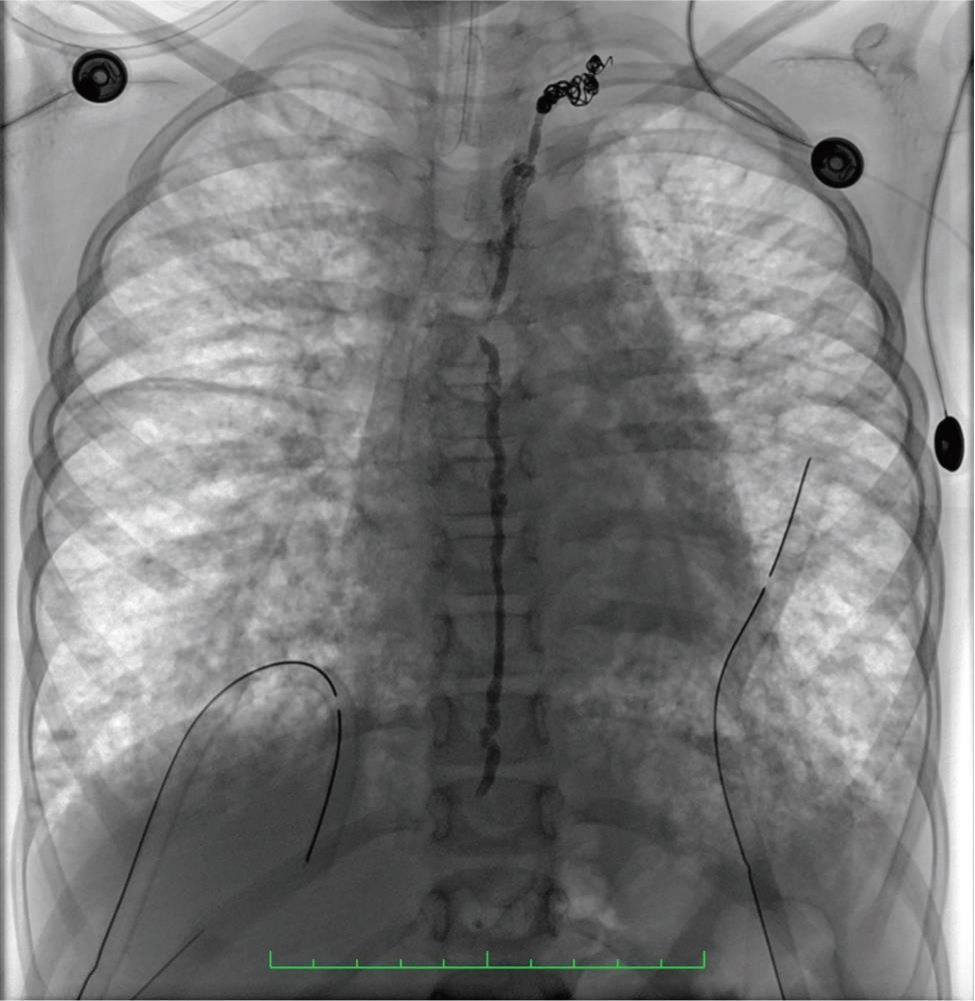
Final frontal angiographic image showing complete embolization of the thoracic duct with detachable coils and a 50% mixture of glue and lipiodol.

Lymphography confirmed TD patency, LM diagnosis, and localized lymphatic leakage into the pericardial cavity (Figure 6). The next passages were conducted according to previously reported experience with selective embolization of the TD [7, 8]. The microcatheter was exchanged for a Renegade STC microcatheter and embolization was performed using Concerto and MicroNester coils, followed by injection of n‐butyl‐2‐cyanoacrylate diluted 1:1 with lipiodol [7, 8]. Final imaging confirmed stasis of the embolizing agent within the TD extending to L1 vertebral level (Figure 7).

## 3. Discussion

This case demonstrates the central value of advanced lymphatic imaging, particularly DCMRL, in the diagnosis and interventional management of complicated thoracic LM. It also highlights the superior efficacy and feasibility of this technique for diagnosing and characterizing LM compared with other imaging modalities, such as lymphoscintigraphy or lympho‐CT [1, 4, 5, 9].

It is important to acknowledge that the therapeutic approach to thoracic lymphatic disorders has evolved over the last decade. Although selective TD embolization has been established as a safe and effective treatment for refractory chylous leaks, increasing evidence suggests that preservation of TD patency should be pursued whenever technically feasible, particularly in patients with congenital lymphatic disorders [7, 10]. The TD represents the main lymphatic drainage pathway, and its occlusion may modify lymphatic hemodynamics, potentially causing development of collateral pathways, lymphatic hypertension, or other recurrent lymphatic complications [8, 10, 11]. Consequently, new interventional strategies—such as selective leak embolization, lymphatic flow diversion, TD stenting—have been increasingly promoted to restore physiological lymphatic drainage while treating the leakage [7, 8, 10, 12]. Nevertheless, TD embolization remains a valuable therapeutic option in selected patients in whom selective interventions are not technically feasible or are unlikely to achieve adequate control of the leak [8, 10, 12].

In our patient, TD embolization resulted in effective control of chylous effusions and clinical improvement, supporting its role as a feasible therapeutic alternative in selected pediatric patients with complex LM. However, current evidence supports the preservation of TD whenever it is possible, and therefore, treatment strategies should be targeted according to the anatomy of the lymphatic system and the eventual lymphatic leakage [2, 7, 9].

## Funding

No funding was received for this manuscript.

## Conflicts of Interest

The authors declare no conflicts of interest.

## Data Availability

Data sharing is not applicable to this article as no datasets were generated or analyzed during the current study.
